# Differential splenic responses to hyperoxic breathing at high altitude in Sherpa and lowlanders

**DOI:** 10.1113/EP091579

**Published:** 2024-01-05

**Authors:** Pontus K. Holmström, Taylor S. Harman, Anne Kalker, Bethany Steiner, Ella Hawkins, Kelsey C. Jorgensen, Kimberly T. Zhu, Ajaya J. Kunwar, Nilam Thakur, Sunil Dhungel, Nima Sherpa, Trevor A. Day, Erika K. Schagatay, Abigail W. Bigham, Tom D. Brutsaert

**Affiliations:** ^1^ Department of Health Sciences Mid‐Sweden University Östersund Sweden; ^2^ Department of Exercise Science Syracuse University Syracuse New York USA; ^3^ Department of Anthropology Syracuse University Syracuse New York USA; ^4^ Department of Anesthesiology Radboud Medical Center Nijmegen Netherlands; ^5^ Department of Anthropology University of California Los Angeles California USA; ^6^ Kathmandu Center for Genomics and Research Laboratory Global Hospital, Gwarko Lalitpur Nepal; ^7^ College of Medicine Nepalese Army Institute of Health Sciences Kathmandu Nepal; ^8^ Local collaborator without institutional affiliation; ^9^ Department of Biology Faculty of Science and Technology Mount Royal University Calgary AB Canada

**Keywords:** acclimatization, adaptation, high altitude, hypoxia, Sherpa, spleen function

## Abstract

The human spleen contracts in response to stress‐induced catecholamine secretion, resulting in a temporary rise in haemoglobin concentration ([Hb]). Recent findings highlighted enhanced splenic response to exercise at high altitude in Sherpa, possibly due to a blunted splenic response to hypoxia. To explore the potential blunted splenic contraction in Sherpas at high altitude, we examined changes in spleen volume during hyperoxic breathing, comparing acclimatized Sherpa with acclimatized individuals of lowland ancestry. Our study included 14 non‐Sherpa (7 female) residing at altitude for a mean continuous duration of 3 months and 46 Sherpa (24 female) with an average of 4 years altitude exposure. Participants underwent a hyperoxic breathing test at altitude (4300 m; barrometric pressure = ∼430 torr; $P_{O_{2}}$ = ∼90 torr). Throughout the test, we measured spleen volume using ultrasonography and monitored oxygen saturation ($S_{pO_{2}}$). During rest, Sherpa exhibited larger spleens (226 ± 70 mL) compared to non‐Sherpa (165 ± 34 mL; *P* < 0.001; effect size (ES) = 0.95, 95% CI: 0.3–1.6). In response to hyperoxia, non‐Sherpa demonstrated 22 ± 12% increase in spleen size (35 ± 17 mL, 95% CI: 20.7–48.9; *P* < 0.001; ES = 1.8, 95% CI: 0.93–2.66), while spleen size remained unchanged in Sherpa (−2 ± 13 mL, 95% CI: −2.4 to 7.3; *P* = 0.640; ES = 0.18, 95% CI: −0.10 to 0.47). Our findings suggest that Sherpa and non‐Sherpas of lowland ancestry exhibit distinct variations in spleen volume during hyperoxia at high altitude, potentially indicating two distinct splenic functions. In Sherpa, this phenomenon may signify a diminished splenic response to altitude‐related hypoxia at rest, potentially contributing to enhanced splenic contractions during physical stress. Conversely, non‐Sherpa experienced a transient increase in spleen size during hyperoxia, indicating an active tonic contraction, which may influence early altitude acclimatization in lowlanders by raising [Hb].

## Abstract (highlights)

- **What is the central question of this study?**
  Indigenous Sherpa exhibit greater spleen size and enhanced exercise‐induced contraction at high altitude compared to lowlanders: how does spleen size of Sherpa and lowlanders respond to hyperoxic breathing at high altitude?
- **What is the main finding and its importance?**
  Sherpa do not experience any changes in spleen size when subjected to hyperoxic breathing at high altitude, whereas lowlanders show a 20% increase in spleen size under similar conditions. This may represent adaptive traits in Sherpa, potentially enhancing the spleen's capacity for contractions during increased metabolic demands.

## 1 INTRODUCTION

The Sherpa residing in the high altitude regions of the Nepal Himalaya are the direct descendants of nomadic Tibetans who migrated south from the eastern Tibetan plateau (Bhandari et al., 2017). They have been residing at high altitude for hundreds of generations (Zhang et al., 2018), sufficient for natural selection to occur, allowing an array of beneficial adaptations to evolve, and enhancing the tolerance to environmentally induced hypoxia (Beall, 2006; Moore, 2017a; Wu & Kayser, 2006). The ease with which high‐altitude‐resident populations, and Sherpa specifically, outperform their lowland counterparts at altitude has become iconic, and is empirically supported by studies showing superior exercise performance and greater maximal oxygen (O_2_) consumption (${\dot{V}}_{O_{2}\max}$) (Brutsaert, 2008; Gilbert‐Kawai et al., 2014). Based on studies in other highland native populations, there is reason to suggest that superior work capacity at high altitude has a genetic basis (Brutsaert et al., 2003). Tibetan‐derived populations, including Sherpa, also have lower (non‐elevated) haemoglobin concentration ([Hb]) at altitude compared to Andeans and acclimatized lowlanders (Beall et al., 1998; Garruto et al., 2003; Moore, 2017b; Wu et al., 2005), and one study of Tibetans showed that participants with lower [Hb] had higher exercise capacity in hypoxia compared to those with higher [Hb] (Simonson, 2015). Thus, conceivably, low [Hb] allows Sherpa to utilize their greater Hb mass (Stembridge et al., 2019) for efficient O_2_ delivery without the adverse effects of high blood viscosity.

In addition to already established phenotypes, Sherpa have recently been observed to have a greater spleen size and enhanced splenic contraction (Brutsaert et al., 2024, Unpublished raw data; Holmström, Mulder, et al., 2020), which may be additional adaptations that have evolved in the Sherpa to increase hypoxia tolerance and aerobic capacity at altitude via enhanced O_2_ carrying capacity. The human spleen stores ∼10% of the total red blood cells (RBC) volume (Stewart & McKenzie, 2002), which can instantly be introduced into systemic circulation in response to various stressors (Shephard, 2016). Typically, the spleen responds to all forms of stress where catecholamines are secreted, including exercise (Holmström et al., 2021; Laub et al., 1993; Sandler et al., 1984; Stewart et al., 2003), voluntary apnoea (Bakovic et al., 2003; Bouten et al., 2019; Schagatay et al., 2001, 2005, 2012), normobaric hypoxia (Lodin‐Sundström & Schagatay, 2010; Richardson et al., 2008), and hypobaric hypoxia (Holmström, Bird, et al., 2020; Schagatay, Holmström, et al., 2020). The result is a splenic contraction that transiently elevates circulating RBCs, which following apnoea results in [Hb] elevations of ∼2–5%, or 0.3–0.6 g/dl (Baković et al., 2005; Bouten et al., 2019; Engan et al., 2013; Holmström et al., 2021; Richardson et al., 2005; Schagatay et al., 2001, 2005, 2007). Although the increase in [Hb] is typically more pronounced after exercise, ranging from 4% to 11% (0.5–1.6 g/dl) (Holmström et al., 2021; Laub et al., 1993), this is generally associated with plasma contractions, which effectively contribute to the change in [Hb]. Accordingly, human splenic contraction may be a protective response against acute and chronic hypoxia via the elevation of O_2_ carrying capacity.

Previously our group found greater spleen size and splenic contraction in response to apnoea and exercise in Sherpa compared to both acclimatized and non‐acclimatized lowlanders (Brutsaert et al., 2024, Unpublished raw data; Holmström, Mulder, et al., 2020). In the first study we observed greater spleen size in Sherpa at low altitude compared with both Sherpa who had migrated to low altitude (20%) and with native lowlanders (35%) at low altitude (Holmström, Mulder, et al., 2020). Larger spleens in Sherpa are in accord with earlier observations of enlarged spleen size in indigenous Bajau, who have been freediving for millennia, compared with a genetically similar land‐based population (Ilardo et al., 2018). It is therefore conceivable that Sherpa and Bajau may have similar benefits from the ability to adjust [Hb] transiently via splenic contraction to cope with hypoxia and regulate blood viscosity. In the same study, we also found that the Sherpa had five‐fold greater splenic contraction in response to apnoea compared with the lowlanders at low altitude, but similar in magnitude compared to Sherpa living at low altitude, suggesting that the splenic contraction of Sherpa holds a functional benefit that is preserved following migration to low altitude. In a more recent study from our group with currently unpublished data, we again found larger spleen size and splenic contraction in Sherpa compared to lowlanders. In this study the Sherpa were acclimatized and studied at high altitude (4300 m), and splenic contraction was induced by intense aerobic exercise instead of via apnoea (Brutsaert et al., 2024, Unpublished raw data). The exercise‐induced contraction of Sherpa was two‐fold greater than acclimatized Nepali non‐Sherpa, potentially linking the Sherpas enhanced spleen size and contraction to their exercise capacity at altitude. While the function of the spleen in Sherpa remains unclear, spleen size and contraction could potentially contribute to O_2_ homeostasis by (1) facilitating transiently increased O_2_ carrying capacity during exercise and (2) maintaining a low [Hb] at rest, thereby keeping blood viscosity low by sequestrating circulating RBCs in the spleen when not needed.

Interestingly, the resting spleen size of the Sherpa, measured at low and high altitude was fairly similar, suggesting that Sherpa spleens in the resting state are unaffected by high altitude exposure (198 ± 56 vs. 234 ± 63 mL). This is not likely the case for lowlanders as a number of human and animal studies suggest that spleens contract immediately on exposure to acute hypoxia, and indeed may stay partially contracted (tonic contraction) in response to chronic hypoxia. In other words, Sherpa spleens may show a ‘blunted’ contractile response to acute and/or chronic hypoxia, despite showing very robust splenic contraction in response to the more severe stimuli of apnoea or exercise in hypoxia. This would be logical, as chronic hypoxia‐induced tonic contraction would limit somewhat the possibility of a full dynamic regulation of [Hb] which we hypothesize to be at the centre of the Sherpa adaptive response. Instead, it would likely be more beneficial for the Sherpa to have a blunted tonic splenic contraction in response to chronic hypoxia, allowing greater transient contractions in situations of increased stress, for example, during bouts of exercise.

In contrast, we had reason to speculate that lowlanders acclimatized to high altitude would not exhibit a blunting of their splenic contraction in response to chronic hypoxia. We recently showed that spleen size decreases with incremental ascent to high altitude in non‐acclimatized native lowlanders, with ∼14% per 1000 m of ascent, which we ascribed to a tonic splenic contraction (Holmström, Bird, et al., 2020). The tonic contraction was also inversely related with the lowlanders’ resting [Hb] throughout the sojourn, further indicating the utility of a tonic splenic contraction during early altitude ascent in lowlanders. While plasma volume reductions are considered the primary factor elevating [Hb] during early high altitude ascent (Siebenmann et al., 2017), a tonic splenic contraction may, in addition, contribute to early haematological acclimatization by elevating [Hb] before the effects of erythropoietin raise [Hb] via erythropoiesis. However, while we found evidence that spleen size is reduced at high altitude in native lowlanders, we could not clearly demonstrate the presence of an active tonic splenic contraction. It is possible that other factors associated with high‐altitude ascent influenced the spleen size reductions. For example, both plasma volume and nitric oxide change with ascent to high altitude (Beall et al., 2012; West, 2004), both of which have been found to influence spleen size at sea‐level (Engan et al., 2020; Toghill & Green, 1972).

Therefore, to further investigate the presence/absence of a blunted splenic contraction versus an active tonic contraction in Sherpa and lowlanders, we measured the spleen volume changes during a short period of hyperoxic breathing in acclimatized participants at 4300 m. We hypothesized that the spleen size of Sherpa, due to a normal blunted response to hypoxia, would be unchanged with the transient relief of hypoxia (i.e., hyperoxic air breathing at 4300 m). In contrast, we hypothesized that the spleen size of acclimatized lowlanders would increase with the transient relief of hypoxia, due to a relaxation of tonic splenic contraction during hyperoxic air breathing.

## 2 METHODS

### 2.1 Ethical approval and participant recruitment

A group of 60 healthy human participants (Table 1) were included; 14 Nepali non‐Sherpa (7 females and 7 males), and 46 indigenous Sherpa (24 females and 22 males). The participants were recruited via convenience sampling, primarily by a local collaborator, either in person or over the phone. All participants underwent a health screening by a medical doctor prior to participation. Because of concerns with literacy in the population, we obtained oral consent from participants after a research investigator read from a script describing the study. Translation and/or clarification was provided by a local collaborator. Most Sherpa participants (*n* = 42) were born at high altitude (≥2500 m), though some (*n* = 4) were born below 2000 m. All Sherpa self‐reported as being Sherpa, whereas the Nepali non‐Sherpa sample self‐reported as Rai (*n* = 8), Tamang (*n* = 5) and Chhetri (*n* = 1), indicating lowland ancestry. The study protocol was approved in advance by the Institutional Review Boards at Syracuse University and the University of California, Los Angeles (IRB: nos 22‐364 and 23‐5041, respectively). We also had local approval by the Nepal Health Research Council (NHRC: no. 4080), which governs biomedical research in Nepal. This study abided by the *Declaration of Helsinki*, except for registration in a data base.

**TABLE 1:** Participants’ anthropometric and demographic data.

	Nepali non‐Sherpa		Sherpa		*P*	ES
	Female (*n* = 7)	Male (*n* = 7)	Female (*n* = 24)	Male (*n* = 22)		
Age (years)	21 ± 3^**^	25 ± 5	28 ± 5	26 ± 6	0.022	0.7
Height (cm)	148.0 ± 4.4^**^	162.0 ± 4.4^*^	154.5 ± 4.5	168 ± 5.0	0.035	0.7
Weight (kg)	51.4 ± 3.2^**^	58.1 ± 10.5	58.3 ± 7.4	66.4 ± 10.5	0.026	0.7
BMI (kg/m^2^)	23.6 ± 0.8	22.1 ± 3.1	24.8 ± 3.6	23.6 ± 3.8	0.244	0.3
FEV_1_ (l)	2.2 ± 0.6	3.0 ± 0.6	2.2 ± 0.6	3.0 ± 1.0	0.757	0.1
FVC (l)	2.6 ± 0.7	3.8 ± 0.3	3.0 ± 0.9	3.9 ± 1.0	0.536	0.2
[Hb] (g/dl)	15.2 ± 1.2	18.0 ± 1.4	15.2 ± 0.9	17.1 ± 1.4	0.316	0.3
Birth altitude (m)	1948 ± 274^***^	1734 ± 301^***^	3409 ± 935	3818 ± 178	<0.001	2.7

*Note*: Data are means ± SD. Data were collected at an altitude of 4300 m, from a study population including indigenous high‐altitude Sherpa (*n* = 46) and Nepali non‐Sherpa (*n* = 14). Significantly different from Sherpa (same sex): ^*^ *P* < 0.05, ^**^ *P* < 0.01, ^***^ *P* < 0.001. Abbreviations: ES, effect size; FEV_1_, forced expired volume in one second; FVC, forced vital capacity; [Hb], haemoglobin concentration.

The primary gateway to the Khumbu valley is Lukla Airport, situated at an elevation of 2860 m. For the purpose of delineating the start of hypoxia exposure for participants who may have been en route from lower regions or residing in Kathmandu for an extended period, we adopted the altitude of Lukla.

Sherpa participants had a relatively high mean continuous altitude exposure time, averaging around 4 years. This was primarily due to the fact that one‐third of the Sherpa participants rarely ventured to lowland areas or had spent their entire lives at high altitude. In contrast, the Nepali non‐Sherpa group had a mean continuous altitude exposure of approximately 3 months, with some individuals having up to 24 months of exposure. Many in this research group were seasonal labourers in Pheriche. Virtually all participants, both Sherpa and Nepali non‐Sherpa, had been at high altitude for at least 1 month before undergoing measurements in Pheriche. An exception was a female Nepali non‐Sherpa participant who had trekked up to Pheriche over a 7‐day period from an altitude of approximately 2003 m. Additionally, three male participants had travelled to Pheriche from regions below 2860 m, with transit times ranging from 6 to 21 days before the study.

All Nepali non‐Sherpa participants had spent the entire week leading up to the study in Pheriche, except for the aforementioned female participant who had transited from an altitude of ∼2003 m to Pheriche over the preceding week. In contrast, Sherpa participants arrived in Pheriche from surrounding areas, including places at higher elevations than Pheriche. Approximately 80% of Sherpa participants came from Pheriche itself or nearby villages ranging from 3400 to 4410 m. Among the remaining Sherpa, 11 individuals had spent time during the week prior at higher altitudes than Pheriche, including transit stops on the Everest Base Camp route and, in some cases, at Everest Base Camp itself, which stands at 5364 m (with six participants having such experiences). Notably, the majority of these 11 individuals were men, while only two Sherpa women had substantial exposure to altitudes exceeding that of Pheriche in the week preceding the study. As mentioned earlier, at least one Sherpa male participant had spent the week prior to the study trekking from Lukla (2860 m) to Pheriche.

### 2.2 Experimental protocol

This experiment was part of a research expedition in the Nepal Himalaya in the town of Pheriche. The experimental design consisted of a hyperoxic breathing test, performed in ambient hypoxia (altitude = 4300 m; barrometric pressure = ∼430 torr; ambient $P_{O_{2}}$ = ∼90 torr). Participants arrived at the temporary field‐laboratory in Pheriche, at which height (cm), body mass (kg) and lung function (forced vital capacity (FVC), forced expiratory volume in 1 s (FEV1)) were measured and age, sex, altitude of birth and ethnicity were gathered via a questionnaire.

### 2.3 Lung function testing

Pulmonary function testing (spirometry) was conducted using a MicroGP portable spirometer (MicroDirect, San Diego, CA, USA). Participants were instructed to inhale maximally, and exhale as quickly and forcefully as possible. Spirometry was performed a minimum of three times, with extra repetitions added if necessary. The maximal values produced were used as the individual's FEV1 and FVC.

### 2.4 Hyperoxia test

The hyperoxia test was performed with participants sitting in a chair. The test started with a 15‐min rest (hypoxia baseline), during which a venous blood sample was drawn during the first 5‐min period. The participants were connected to a breathing valve Douglas bag set‐up that allowed switching from room air to hyperoxia ($F_{iO_{2}}$ = 0.35, balance N_2_). This level of hyperoxia was designed to ‘return’ participants to sea‐level conditions. Ten minutes before hyperoxia, participants were instrumented with a mouthpiece and breathed ambient room air, and $S_{pO_{2}}$ measurements were started. Following 10 min of breathing room air, a nose clip was attached, and the breathing valve was switched to O_2_‐enriched air (hyperoxia) from the Douglas bag. The hyperoxia intervention lasted 5 min, after which the breathing valve was switched back to room air and the participants rested for an additional 5 min (hypoxia recovery).

### 2.5 Splenic ultrasound measurements

**FIGURE 1:**
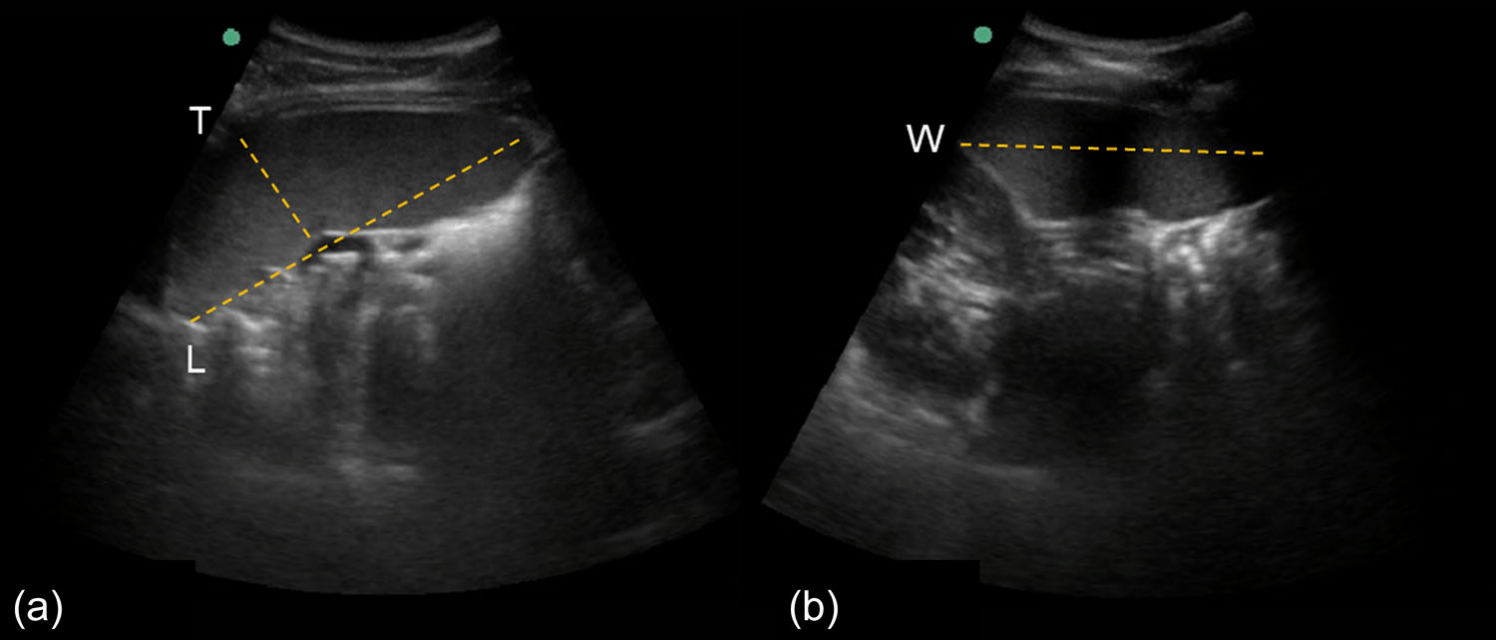
Spleen measurements were collected from the left dorsal side in the longitudinal and transverse plane from three axial splenic diameters of maximal splenic length (*L*) and thickness at hilum (*T*) (a) and width (*W*) (b).

The spleen was measured from the left dorsal side via ultra‐sonic imaging (M‐Turbo Ultrasound system, Fujifilm SonoSite Inc., Bothell, WA, USA) with the probe: C60x/5−2 MHz (SonoSite Inc.), by an experienced sonographer (P.H.). Two ultrasonic images were collected each minute for determination of the maximal three‐axial diameters: length (*L*), thickness (*T*; Figure 1a) and width (*W*; Figure 1b). The baseline (resting) spleen volume was measured for 5 min during the last 5 min of the 10‐min rest phase before hyperoxic gas was administered (hypoxic baseline). In addition, spleen measurements were collected continuously for 5 min during the hyperoxia and for 5 min immediately following the hyperoxia during the recovery phase (hypoxic recovery).

### 2.6 Peripheral oxygenation measurements

Peripheral O_2_ saturation ($S_{pO_{2}}$) was recorded continuously on a second‐by‐second basis, throughout the test using a peripheral pulse oximeter (Medair Lifesense LS1‐9R, Medair AB, Delsbo, Sweden) attached to the index finger. Data were stored via a memory unit (Trendsense, Nonin Medical Inc., Medair AB, Hudiksvall, Sweden) for subsequent analysis.

### 2.7 Hematological measurements

To obtain [Hb] measures, single venous blood samples were collected from an antecubital arm or hand vein by a physician. Due to challenges identifying an appropriate vein to puncture, the procedure of collecting blood samples occurred over approximately 3–4 min, prior to collecting baseline splenic measurements. [Hb] measurements were analysed in duplicate and analysed immediately via a haemoglobinometer (HemoCue Hb 201 microcuvettes, Ängelholm, Sweden).

### 2.8 Analysis

Measurements of the maximal splenic length (*L*), thickness (*T*), and width (*W*) were used to calculate spleen volume according to the Pilström equation (Schagatay et al., 2005):

$$V_{\mathrm{spleen}}=L\pi \left(WT-T^{2}\right)/3$$

The equation describes the difference between two ellipsoids and has been used in similar experiments involving spleen volume assessments in association with apnoea and exercise (Engan et al., 2014; Holmström et al., 2021; Schagatay et al., 2012) and is associated with high reliability between repeated tests (Holmström et al., 2022). Baseline (resting) spleen volume was obtained from the 5‐min period before the hyperoxia by averaging the five splenic measurements collected (Holmström et al., 2022). Thereafter, hyperoxic spleen volume was assessed and determined at the point $S_{pO_{2}}$ increased above 98% saturation, which occurred in both groups by the fourth minute. Therefore, the spleen volume measurements obtained the fourth and fifth minute into the hyperoxia were averaged and used as a representation of hyperoxia‐induced spleen volume. Spleen volume change was then calculated as an absolute (ml) and relative (%) change from baseline to the hyperoxia‐induced spleen volume. Spleen volume was expressed in millilitres and in relation to body height (ml/cm).

From the hypoxia baseline, the second‐by‐second $S_{pO_{2}}$ measurements were averaged on a per minute basis, yielding five $S_{pO_{2}}$ values that were then averaged and used to define baseline $S_{pO_{2}}$. The same principle, average on a per minute basis, was also applied to analyse $S_{pO_{2}}$ values during hyperoxia and hypoxic recovery. Technical difficulties during the data collection resulted in missing $S_{pO_{2}}$ values of one participant effectively precluding $S_{pO_{2}}$ data for the full non‐Sherpa sample (*n* = 13).

### 2.9 Statistical analysis

Data are presented as means ± SD unless otherwise stated. All statistical tests were carried out using IBM SPSS 24.0 for Windows (IBM Corp., Armonk, NY, USA). The level of significance was set at *P* < 0.05. The Shapiro–Wilk test was used to assess whether study variables were normally distributed. Assumptions of homoscedasticity of the data were assessed with Levine's test of equal variance. A 2 × 3 mixed ANOVA was conducted to evaluate the effects of sex (male/female) and high altitude exposure (Sherpa/Nepali non‐Sherpa) on spleen volume change (dependent variable). Assessments of within‐group differences on spleen volume changes were conducted using a one‐way repeated‐measures ANOVA with Bonferroni adjustments for multiple comparisons. Differences between Sherpa and Nepali non‐Sherpa were assessed using Student's independent samples *t*‐test, while Student's paired samples *t*‐test was conducted to assess within‐subject differences on responses induced by hyperoxia. In all group comparisons height was used as a covariate to control for body size. Pearson's product‐moment correlation coefficient (*r*) wase used to quantify associations between parametric variables and Spearman's rho (ρ_S_) was used for non‐parametric variables (sex). The magnitude of observed effects was estimated by the standardized mean difference using Cohen's *d* effect size (ES), computed as the mean difference divided by the pooled SD. ESs are presented with 95% confidence intervals (CI) and an ES of 0.2–0.3 was considered small, 0.4–0.7 as medium, and ≥0.8 as large (Lee, 2016). The ES in Figures 3 and 4 were derived from bootstrapping (Ho et al., 2019).

## 3 RESULTS

There were no interactions of sex‐by‐study group for spleen volume change or $S_{pO_{2}}$ change in response to breathing hyperoxic gas at high altitude. As a consequence, male and female data points were combined for the analysis reported below.

### 3.1 Peripheral oxygen saturation changes in response to hyperoxia

**FIGURE 2:**
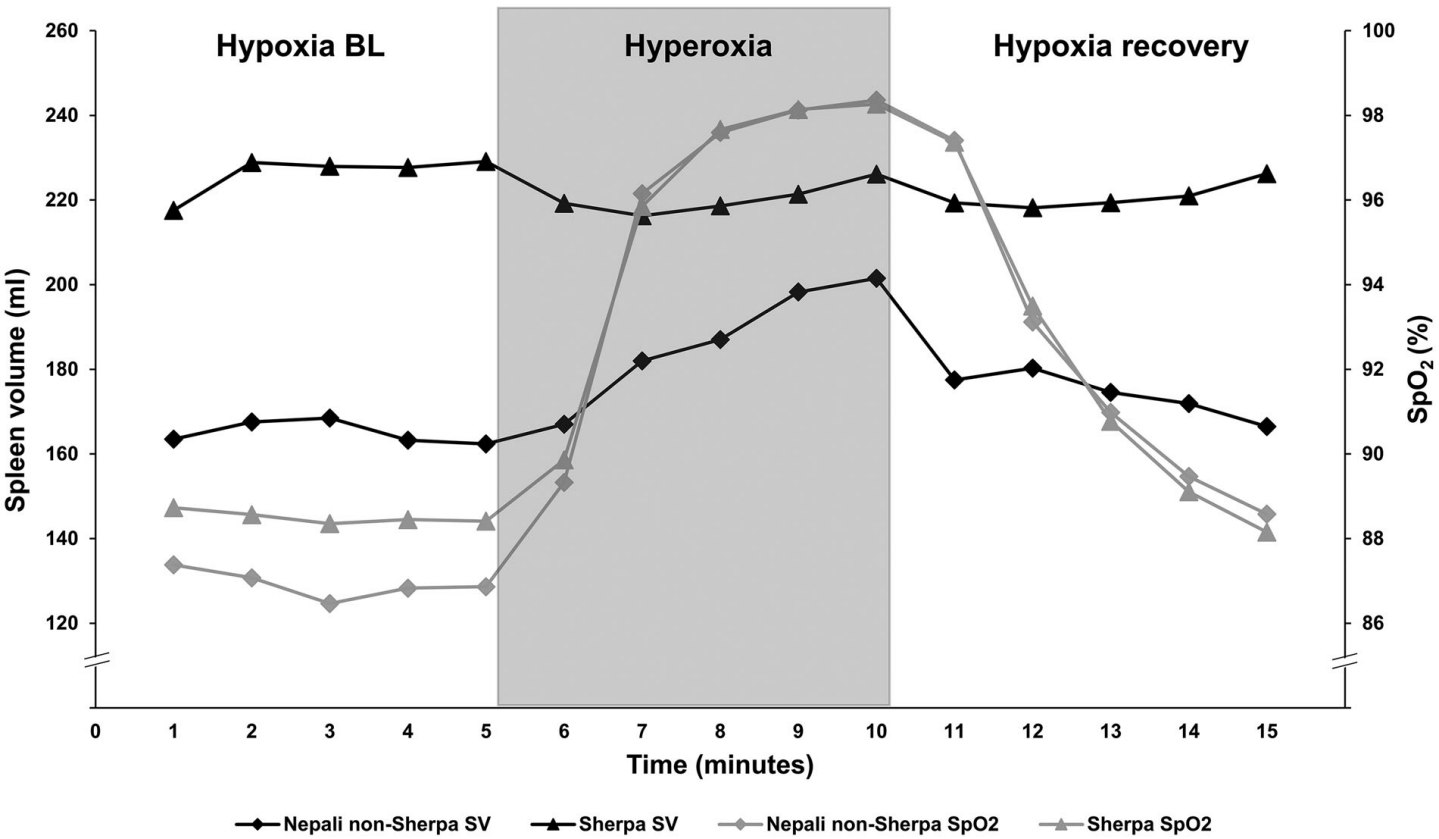
Spleen volume change (mean, left *y*‐axis) and $S_{pO_{2}}$ change (mean, right *y*‐axis) throughout the hyperoxic test, displaying data points for three phases of the test: (1) baseline breathing ambient air in hypoxia at rest (hypoxia BL), (2) breathing O_2_ enriched air (hyperoxia), and (3) recovery breathing ambient air (hypoxia recovery), for Sherpa (*n* = 46, triangles) and Nepali non‐Sherpa (*n* = 14, *n* = 13 for $S_{pO_{2}}$, diamonds).

Baseline $S_{pO_{2}}$ was similar between Sherpa (88.5 ± 4.1%) and Nepali non‐Sherpa (86.9 ± 2.4%, *P* = 0.198, Figure 2). By the fifth minute of hyperoxia, $S_{pO_{2}}$ increased significantly over baseline in both Sherpa (by 9.7%, 95% CI: 8.1–11.3; *P* < 0.001; ES = 2.5, 95% CI: 1.99–3.04) and non‐Sherpa (by 11.4%, 95% CI: 9.5–13.4; *P* < 0.001; ES = 4.8, 95% CI: 2.83–6.78; Figure 2). However, this increase (i.e., change) in $S_{pO_{2}}$ was similar between study groups, that is, there was no group‐by‐time interaction (*F*(2, 114) = 1.754, *P* = 0.178, Figure 2). In both groups, a mean of 98% $S_{pO_{2}}$ was reached by the fourth minute of hyperoxia (*P* < 0.001) and $S_{pO_{2}}$ returned to baseline values following 5 min of breathing ambient air in both Sherpa (88.1 ± 2.4%, *P* = 0.99) and non‐Sherpa (88.5 ± 1.6%, *P* = 0.218).

### 3.2 Spleen size at baseline

**FIGURE 3:**
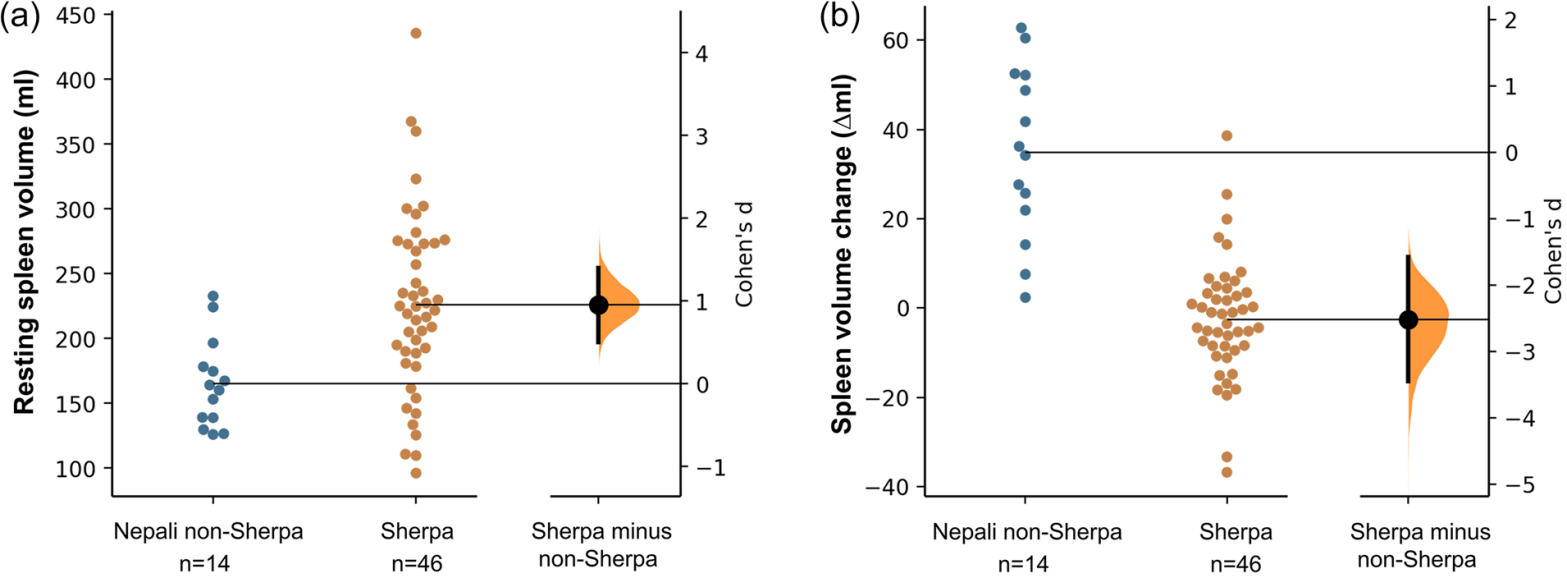
Estimation plot of effect size (ES, Cohen's *d*) between Sherpa (*n* = 46) and Nepali non‐Sherpa (*n* = 14) for (a) baseline spleen volume (ml) and (b) magnitude of spleen volume change (∆ml),) induced by breathing hyperoxic gas in Pheriche (4300 m). Both groups are plotted on the left‐axis with individual Nepali non‐Sherpa marked by turquoise circles and Sherpa by orange circles. The ES, depicted as a black circle with error bars, is plotted on a floating *y*‐axis on the right with its bootstrap sampling distribution.

Resting spleen volume of Sherpa (226 ± 70 mL, 140 ± 39 mL/m) was 37% larger compared with Nepali non‐Sherpa (165 ± 34, 106 ± 21 mL/m; *P* < 0.001; ES = 0.95, 95% CI: 0.3–1.6; Figure 3a), by a mean difference of 61 ± 19 mL (95% CI: 22–100 mL). In addition, baseline spleen volume was not associated with height (*r* = 0.180, *P* = 0.539), weight (*r* = 0.429, *P* = 0.126), sex (ρ_S_ = −0.051, *P* = 0.863) or age (*r* = 0.514, *P* = 0.060) in the non‐Sherpa, likely influenced by the small sample size (*n* = 14). In contrast, in the much larger Sherpa sample (*n* = 46), spleen volume was associated with sex (ρ_S_ = 0.456, *P* = 0.001), height (*r* = 0.600, *P* < 0.001), and weight (*r* = 0.390, *P* = 0.007), but not with age (*r* = −0.018, *P* = 0.907).

### 3.3 Spleen volume changes in response to hyperoxia

**FIGURE 4:**
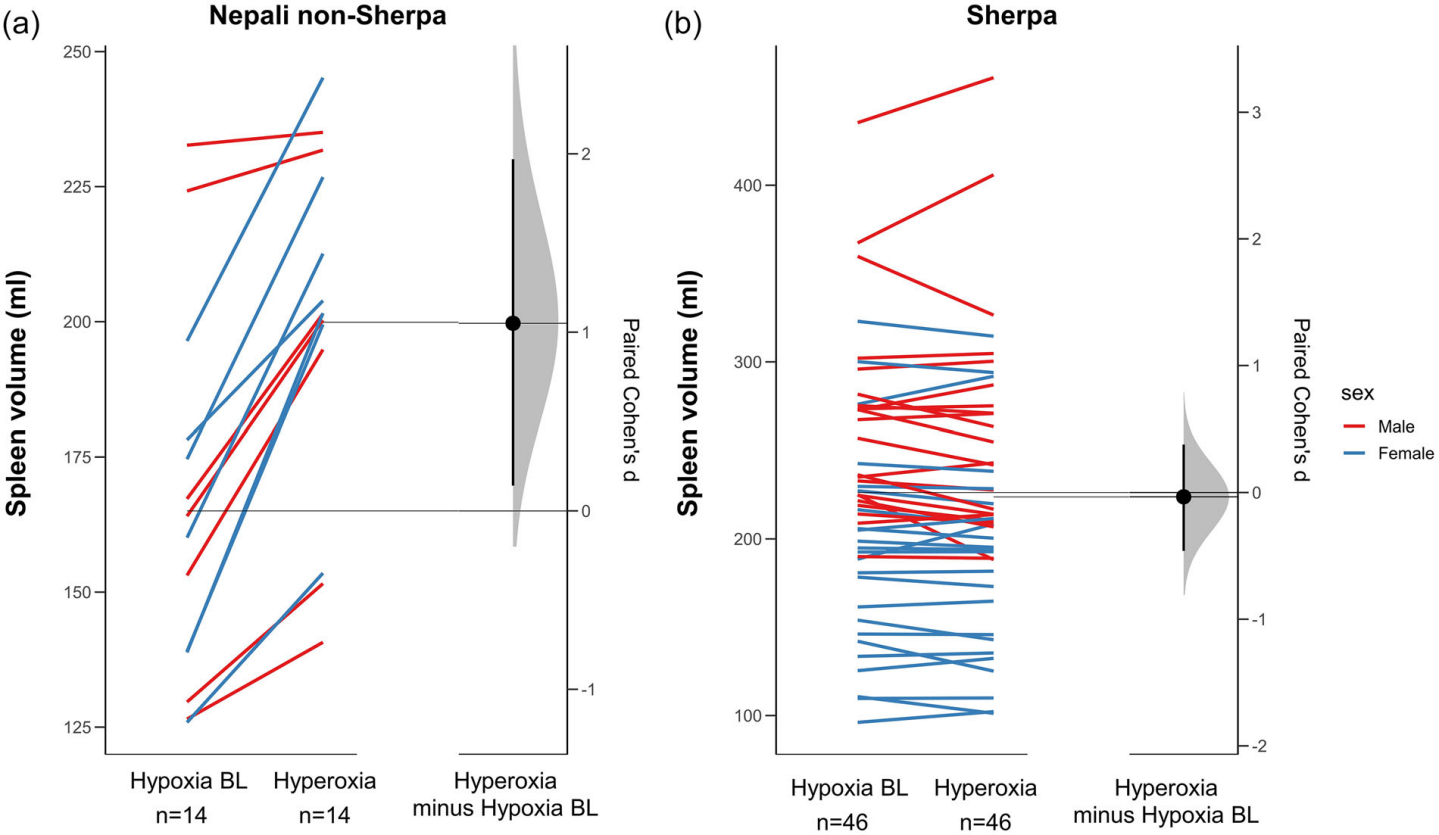
Estimation plot of paired effect size (ES, Cohen's *d*) between baseline (hypoxia BL) and hyperoxia in Nepali non‐Sherpa (a) and Sherpa (b) for spleen volume change (ml) following 5 min of hyperoxia. Both hypoxia BL and hyperoxia are plotted on the left‐axis as a slope graph, with a line (red for the females and blue for males) connecting each paired set of observations. The paired ES are plotted on a floating *y*‐axis on the right as a bootstrap sampling distribution. The ES is depicted as a black circle with 95% confidence intervals indicated by the vertical error bars.

The magnitude of the hyperoxia‐induced spleen volume increase was greater in Nepali non‐Sherpa compared with the Sherpa (mean difference of 37 ± 5 mL, 95% CI: 28.7–46.4; *P* < 0.001; ES = 2.5, 95% CI: 1.75–3.25; Figure 3b). For the non‐Sherpa, spleen volume increased by 22 ± 12% (35 ± 17 mL, 95% CI: 20.7–48.9; *P* < 0.001; ES = 1.8, 95% CI: 0.93–2.66; Figure 3a) in response to hyperoxia, while spleen volume was unchanged for the Sherpa (−2 ± 13 mL, 95% CI: −2.4 to 7.3; *P* = 0.640; ES = 0.18, 95% CI: −0.10 to 0.47; Figure 4b). Similarly, the study groups had disparate change in spleen volume in response to hyperoxia at high altitude, after controlling for height (*F*(2, 114) = 1.549, *P* < 0.001, Figure 2). After 5 min of breathing ambient air, spleen volume was returned to baseline values for the non‐Sherpa (1.5 mL, 95% CI: 7.9–10.9; *P* = 0.99).

**FIGURE 5:**
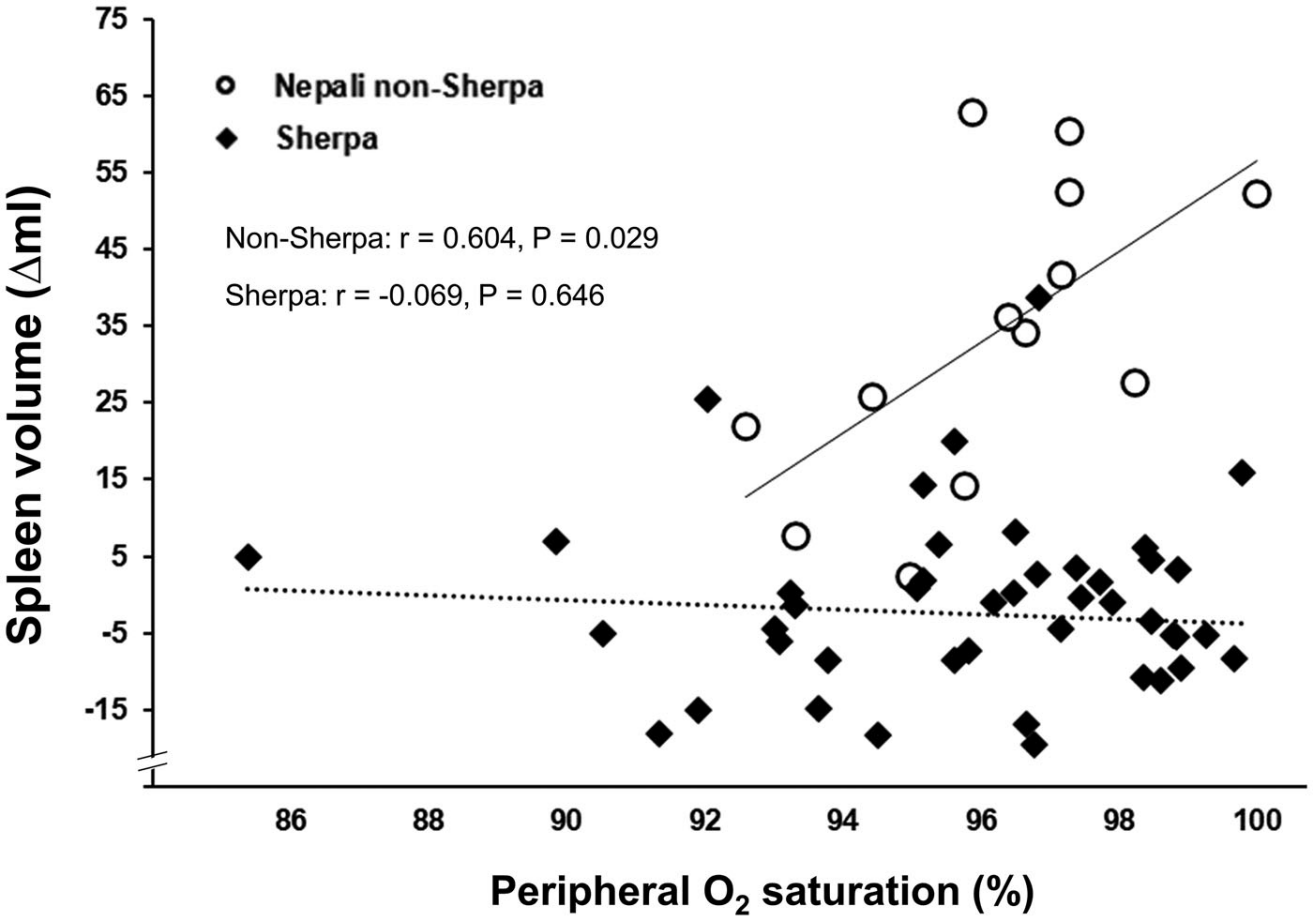
Correlation plots of spleen volume change (∆ml) and peripheral O_2_ saturation ($S_{pO_{2}}$) during the second minute of the hyperoxic episode for Sherpa (*n* = 46) and Nepali non‐Sherpa (*n* = 13). Minute 2 was chosen as an illustration as it marks the time during hyperoxia when $S_{pO_{2}}$ displayed the largest variation and rose from high‐80s to mid‐90s. Filled diamonds indicate Sherpa and open circles indicate Nepali non‐Sherpa.

In order to investigate a putative link between the relief of hypoxia and splenic contraction, an additional correlational analysis was conducted. There was a positive association between the magnitude of the splenic volume increase (i.e., the change in volume from baseline to hyperoxia) and $S_{pO_{2}}$ throughout the duration of hyperoxia in non‐Sherpa (min 1: *r* = 0.031, *P* = 0.921; min 2: *r* = 0.604, *P* = 0.029; min 3: *r* = 0.645, *P* = 0.017; min 4: *r* = 0.554, *P* = 0.05; min 5: *r* = 0.602, *P* = 0.029). No association was found for the Sherpa (not significant, Figure 5).

## 4 DISCUSSION

This is the first study to investigate spleen volume change during hyperoxic breathing at high altitude (4300 m) in indigenous highlander Sherpa compared to a group of Nepali non‐Sherpa with lowland ancestry. The main findings were that: (1) the acclimatized Sherpa exhibited no change in spleen size during hyperoxic breathing, while for the acclimatized non‐Sherpa spleen size increased by more than 20%; (2) the spleen volume increase, induced by hyperoxia, was positively associated with the measured $S_{pO_{2}}$ during hyperoxia in Nepali non‐Sherpa, but not in Sherpa; (3) Sherpa exhibited greater resting spleen size than that of the Nepali non‐Sherpa at high altitude. The observation of differential spleen volume change during hyperoxia at high altitude despite similar $S_{pO_{2}}$ changes in acclimatized Sherpa and acclimatized non‐Sherpa may reflect two distinct patterns of splenic function at high altitude.

### 4.1 Splenic function in Sherpa

One of the primary findings was the observation of unchanged hyperoxia‐induced spleen volume in acclimatized Sherpa, indicating that Sherpa spleens are not influenced by high altitude per se, that is, that tonic splenic contraction is not a feature of Sherpa at high altitude. This makes sense as a constant hypoxia‐induced tonic splenic contraction under normal circumstances would effectively limit the capacity for beneficial splenic contraction during severe hypoxia and/or increased metabolic demand. Instead, it appears more functionally beneficial to have a blunted splenic response at rest in hypoxia, allowing for lowering resting [Hb], and then subsequent greater contractions during transient stressors, like bouts of exercise. In other words, with a lack of a tonic contraction more of the blood cells are sequestered in the spleen, effectively positioning the Sherpa for a large infusion of RBCs when the spleen eventually does contract during increased stress. We recently showed greater exercise‐induced splenic contraction in Sherpa at high altitude compared with acclimatized individuals of lowland ancestry (Brutsaert et al., 2024, Unpublished raw data). This finding substantiates the practical importance of a blunted tonic splenic contraction at high altitude in Sherpa. Accordingly, a blunted splenic response (i.e., resting spleen at its largest capacity) allows greater capacity for contractions compared with lowlanders at high altitude. Lowlanders, unlike Sherpa, are likely not characterized with a blunted contraction, but exhibit a tonic splenic contraction at high altitude; subsequently they have less capacity for additional splenic contractions at high altitude. This agrees with our previous observations of enhanced apnoea‐induced contraction in Sherpa compared with Nepali lowlanders (Holmström, Mulder, et al., 2020), showing that Sherpa have greater splenic contraction in response to a variety of stressors (i.e., exercise and breath‐holding). That the Sherpa have an enhanced ability to contract their spleens appears to be a universal response, rather than isolated to a specific stressor, and likely depended upon the size of the resting spleen, which in turn is influenced by the blunting or relaxation at rest in hypoxia. The observation that Spleen volume change and $S_{pO_{2}}$ during hyperoxia was unrelated in Sherpa may indicate that splenic reactivity in Sherpa is uninfluenced by hypoxia, which further speaks to a blunted splenic response at high altitude.

Recent research indicates that Sherpa have larger plasma volume compared with Andeans and lowlanders at high altitude, effectively masking a larger Hb mass in Sherpa (Stembridge et al., 2019). The lower [Hb] demonstrates a unique adaptation in Sherpa that moves attention away from high [Hb] and increased O_2_ carrying capacity per se, and instead focuses attention on an efficient regulation of circulating RBCs with associated reduced blood viscosity as likely more beneficial. While the functional aspect of splenic contraction in the Sherpa population currently is unknown, it is conceivable that the spleen may contribute to O_2_ homeostasis in Sherpa by on the one hand facilitating transiently enhanced O_2_ carrying capacity during bouts of exercise, via the [Hb] elevating contraction, and on the other hand by maintaining a low [Hb], thus ensuring low blood viscosity, by sequestrating circulating RBCs in the spleen at rest.

Our results also show that indigenous Sherpa have spleens that are ∼37% larger in resting conditions at altitude compared with their Nepali non‐Sherpa counterparts. First, it should be noted that our study groups had been living at high altitude for varying durations. The Sherpa, with a mean exposure time of 4 years, had a longer period at altitude compared to the lowlanders, who had been exposed for an average of 3–12 months. We consider this duration sufficient for potential long‐term effects to manifest in lowlanders. Therefore, it is highly likely that the observed difference in spleen size is not influenced by the varying durations the study groups had resided at high altitude before testing. Larger spleen size in Sherpa parallels recent observations from our group, demonstrating greater spleen size in Sherpa compared with Nepali non‐Sherpa at both low (Holmström, Mulder, et al., 2020) and high altitude (Brutsaert et al., 2024, Unpublished raw data). In both cases, differences between study groups were impressively large (Cohens *d* effect size = 1.4 and 1.2, respectively), and indicate a practical significance of larger spleens in the Sherpa. A recent investigation on competitive freedivers found that top‐tier freedivers had substantially larger spleens (538 mL) compared with their less successful counterparts (270 mL). They ascribed a 15 s gain in apnoea time to the size differences between top‐scoring freedivers and low‐scoring freedivers (Schagatay et al., 2012). This variation highlights two critical points: first, there is a significant variation in spleen sizes among individuals, and second, individuals with smaller spleens may be bereft of any distinct functional advantages. This prompts the need to distinguish between those with sufficiently large spleens and the associated contractility that provides functional benefits, and those with smaller spleens that may lack any functional advantages. This reasoning can be further illustrated by considering changes in $C_{aO_{2}}$ as a consequence of changes in spleen size and contraction. Based on baseline [Hb] of 16.65 g/dl and $S_{pO_{2}}$ of 86.92%, in the lowlander sample, a resting $C_{aO_{2}}$ is assumed to be around 20 mL O_2_/dl blood (range 17.6–22.9 mL/dl). With an addition of 30 mL RBC, $C_{aO_{2}}$ would increase to 20.24 mL/dl (range 17.9–23.2 mL/dl). However, 30 mL is likely an underestimation and an average difference in spleen volume between 1400 and 4300 m is likely closer to 60 mL (Holmström, Mulder, et al., 2020). In that case $C_{aO_{2}}$ would increase to 20.48 (range 18.4–23.5 mL/dl), still a small increase, but perhaps not negligible in the non‐acclimatized lowlander at the upper end of the $C_{aO_{2}}$ range who is in the early stages of acclimatization. In addition, as these simple calculations are based on means, they do not take individual variations into account. In a presumed responder with a resting spleen size of around 300 mL at low altitude and around 150 mL at 4300 m, $C_{aO_{2}}$ would increase to 23.7 mL/dl. An important point here is that the $C_{aO_{2}}$ varies greatly in a sample of acclimatized lowlanders at high altitude, and it is perhaps not unreasonable to assume that individuals with particularly large spleens may have greater influence on $C_{aO_{2}}$ that may confer some practical meaning.

Larger spleen size and contraction have, similarly, been observed in freedivers (Bakovic et al., 2003; Ilardo et al., 2018; Prommer et al., 2007; Schagatay et al., 2012), with contemporary greater apnoea‐induced [Hb] increase in divers compared with non‐divers (Baković et al., 2005; Elia et al., 2019; Richardson et al., 2005). Consequently, spleen size and contraction have been associated with apnoeic durations in both novice and experienced divers (Bouten et al., 2019; Elia et al., 2021) and with overall freediving performance (Schagatay et al., 2012). Therefore, it appears conceivable that similar effects are present at high altitude in the Sherpa population, allowing enhanced tolerance to hypoxia. Nonetheless, the current Sherpa sample averaged 226 mL in spleen size, but with a considerable variation ranging from 96 to 435 mL, possibly suggesting a similar situation in terms of functional benefit in Sherpa as in freedivers as described above.

A previous genomic investigation observed greater spleen size in Bajau divers compared with a closely related non‐diving population and suggested that natural selection on variants in the *PDE10A* gene could be responsible (Ilardo et al., 2018). They also found that non‐diving Bajau had equally large spleens as their diver counterparts, whereby the authors concluded that the larger spleens of the Bajau were not attributed to phenotypic plasticity, but solely to genetic adaptation. While the mechanisms responsible for large spleen size and blunting of the splenic contraction at altitude in Sherpa are unknown, we propose a potential connection with the *PDE10A* gene. *PDE10A* codes for a cyclic nucleotide phosphodiesterase, which plays a role in α‐adrenergic responses and the contraction of smooth muscle such as that surrounding the spleen (Pinkus et al., 1986) and is responsible for splenic contraction (Ilardo et al., 2018). Ilardo et al. (2018) found significant associations between one SNP, rs3008052, in the *PDE10A* gene, with spleen size, and therefore it appears likely that upregulation of rs3008052 also could modify the contraction in response to selected stressors, that is, hypoxia, resulting in blunting of the splenic contraction. Our previous findings revealed larger spleens in Sherpa compared with lowlanders (Brutsaert et al., 2024, Unpublished raw data; Holmström, Mulder, et al., 2020), indicating that increased spleen size in Sherpa may be a genetic adaptive response in line with observations by Ilardo et al. (2018). We also observed larger spleens in Sherpa living at high altitude compared with a group of Sherpa who migrated to low altitude, which may indicate that some splenic adjustments are lost over time when the initial hypoxic stress is eliminated (i.e., de‐acclimatization) (Holmström, Mulder, et al., 2020). Therefore, we suggested that larger spleens in Sherpa could be due to both genetic and environmental factors, and we were unable to rule out the role of developmental adaptation. Whatever the origin of these traits, it is possible that both high altitude Sherpa and Bajau divers share similar benefits from the ability to adjust [Hb] via splenic contraction. Nevertheless, the question of whether evolutionary adaptation has indeed shaped the human spleen at high altitude remains unanswered, emphasizing the need for further research to elucidate the origins of the enlarged spleen size and greater splenic contraction observed in the Sherpa population.

### 4.2 Splenic function in lowlanders

The finding that spleen size increases by over 20% in response to hyperoxic breathing in acclimatized individuals with lowland ancestry indicates that splenic contraction occurs in lowlanders at high altitude, and in contrast to the Sherpa, this contraction is tonic, simply meaning that the contraction is sustained over time with sustained hypoxic stress. We speculate that the tonic contraction in lowlanders may have functional benefit during ascent to high altitude by influencing haematological acclimatization, and by preserving $C_{aO_{2}}$ early in ascent (Holmström, Bird, et al., 2020; Schagatay, Lunde, et al., 2020).

Earlier findings from our group demonstrated that baseline spleen size decreases with incremental ascent to high altitude, with ∼14% per 1000 m of ascent, concurrently elevating circulating [Hb] (Holmström, Bird, et al., 2020). In that study, the participants were non‐acclimatized prior to testing, and slowly acclimatized as the expedition continued and testing altitude increased (Holmström, Bird, et al., 2020). Thus, they were never fully acclimatized, as measurements were collected early at each new altitude up to 5160 m. In the present study, however, the participants were fully acclimatized, with a minimum of 4 weeks spent at altitude before testing. The fact that we observed a hyperoxia‐induced spleen volume increase in apparently fully acclimatized individuals strengthens the evidence of a tonic splenic contraction at high altitude in lowlanders. Even following full acclimatization (weeks) in lowlanders, the spleen is still marginally small, allowing an acute increase in size when the hypoxic stress is relieved (i.e., in response to increased arterial O_2_ saturation). Our observations of a tonic splenic contraction in lowlanders agrees with a recent field research expedition to high altitude, which, similarly, demonstrated a tonic splenic contraction with incremental high altitude ascent in lowlanders (Schagatay, Lunde, et al., 2020).

The rate of erythropoiesis is slow: Hb mass is still relatively unchanged following 8 days at an altitude of ∼4000 m (Siebenmann et al., 2017), indicating that other factors than erythropoiesis determine the [Hb] increase seen during the first few days at altitude, at least up to ∼4000 m. While the initial rise in [Hb] at high altitude is considered to be primarily a result of reductions in plasma volume (Siebenmann et al., 2017), our current and previous findings (Holmström, Bird, et al., 2020; Schagatay, Lunde, et al., 2020) demonstrate that the tonic splenic contraction potentially contributes to some of the observed polycythaemia at high altitude in the earliest stages of exposure, thereby influencing the haematological acclimatization process during early high altitude ascent. While the potential functional benefits of the spleen during the early acclimatization process are intriguing, there currently is limited research evidence of this. Indeed, studies investigating blood infusion and erythropoiesis stimulation during aerobic exercise at high altitude typically report no change in ${\dot{V}}_{O_{2}\max}$, although [Hb] and $C_{aO_{2}}$ are significantly elevated (Lundby & Damsgaard, 2006; Young et al., 1996). A reasonable explanation as to why ${\dot{V}}_{O_{2}\max}$ is uninfluenced by elevated [Hb] at high altitude may be due to O_2_ diffusion limitations and a lack of driving pressure for $P_{aO_{2}}$ into skeletal muscle tissues. Although the pathway underpinning a putative increase in ${\dot{V}}_{O_{2}\max}$ as a result of exercise‐induced splenic contraction is unclear, high altitude ascent and subsequent acclimatization typically occur at sub‐maximal intensities (Pugh, 1958). During two recent expeditions to high altitude, we observed elevated $C_{aO_{2}}$ following apnoea (Holmström, Bird, et al., 2020) and an association between resting spleen size and development of acute mountain sickness during incremental ascent to 4400 m (Holmström et al., 2019), suggesting that splenic contraction during early high altitude ascent may elevate $C_{aO_{2}}$ and attenuate the development of acute mountain sickness symptoms.

We also observed a positive association between the change (i.e., increase) in spleen volume and $S_{pO_{2}}$ in Nepali non‐Sherpa, which was particularly prominent early in the hyperoxic episode before $S_{pO_{2}}$ had stabilized around 98%. This may or may not be causal and the sample size was low, but this association suggests that levels of $S_{pO_{2}}$ could be directly linked to spleen volume change. During hyperoxic breathing and the transient relief of hypoxia, individuals with an early rise in $S_{pO_{2}}$ exhibited a greater spleen volume increase, and vice versa. This could be due to a direct link between the level of systemic hypoxia and the magnitude of tonic splenic contraction in lowlanders exposed to hypoxia. The link between the tonic splenic contraction and $S_{pO_{2}}$ is consistent with previous research demonstrating that hypoxia is the primary initiating mechanism for the splenic contraction. Splenic contraction has been observed during short‐term normobaric hypoxia exposure, as was previously described (Lodin‐Sundström & Schagatay, 2010; Richardson et al., 2008). In addition, we previously observed an association between the tonic splenic contraction and resting arterial O_2_ saturation during incremental ascent to high altitude (Holmström, Bird, et al., 2020). That particular association could also explain our observation that individuals with lower $S_{pO_{2}}$ during incremental high altitude also had smaller spleens during ascent, (i.e., these individuals experienced greater tonic contraction throughout ascent at high altitude compared to those with higher $S_{pO_{2}}$). Taken together, these findings provide an argument that the tonic splenic response in lowlanders at high altitude is initiated directly by systemic hypoxia. In other words, in lowlanders, the tonic splenic contraction appears to be active rather than passive or indirect (i.e., indirectly influenced by other factors present at high altitude and not hypoxia per se).

In contrast to the observations of the current study, an earlier investigation failed to discern evidence of splenic contraction at high altitude with potential functional benefits in lowlanders, in that they found that spleen size was unchanged with incremental ascent to high altitude (Purdy et al., 2018). Nonetheless, it is essential to consider several significant factors. Firstly, the maximum altitude of 4240 m at which spleen measurements were taken in their study is notably lower than the 5160 m altitude that we more recently utilized (Holmström, Bird, et al., 2020). This distinction in maximal altitude could have significant physiological implications since higher altitudes induce more severe hypoxia and potentially lead to more pronounced splenic contractions. Additionally, it is worth highlighting that the baseline spleen volume measurements in their study displayed an unusual degree of variation under otherwise similar control conditions. This variation raises concerns about potential high variability between baseline conditions and altitude conditions, which could ultimately impact the statistical analysis, especially in a study with only 12 participants. Furthermore, methodological variations are noteworthy. In particular, Purdy et al. (2018) employed a distinct calculation method to estimate spleen volume, which tends to overestimate volume measures in comparison to the equation used in our current analysis. Moreover, a fundamental distinction lies in the number of spleen measurements collected during baseline, which is critical for calculating an accurate spleen volume. While our approach involved a 5‐min baseline period with measurements taken every minute, Purdy et al. (2018) employed a 3‐min baseline period with only two measurements. These seemingly minor methodological differences could potentially have had substantial impact on measurements, particularly given the existing degree of high variation.

### 4.3 Limitations

There are a number of limitations associated with the current data collection that require acknowledgement. First, a challenge with ultrasonography for determining spleen size is the potential measurement error resulting from subjective identification of relevant landmarks and biological variation associated with pulsatile volume changes (Cesta, 2006). Nevertheless, a recent investigation demonstrated that our method is associated with high reliability, reflected by a low coefficient of variation (2.98 ± 0.1%) and high intraclass correlation coefficient (0.970, *P* < 0.001) between repeated tests (Holmström et al., 2022). Additionally, spleen size measured by ultrasound exhibit strong correlations with actual spleen size measured from cadavers (Loftus et al., 1999), which further supports ultrasonography for spleen size assessments. A concern that arises from this subjective identification of spleen size in between‐group comparisons is potential measurement error associated with bias of the sonographer. Therefore, an important consideration concerns blinding the sonographer to study group membership to mitigate potential human measurement errors. However, the current study did not include blinding during the data collection. Our data collection protocol involves immediate collection and analysis of spleen volume measurements, and sacrifices blinding but offers other advantages. It allows the sonographer to assess a dynamic, real‐time representation of the spleen's changes over time, a crucial aspect of ultrasonography for understanding the unique characteristics of each spleen, which is naturally associated with high pulsatile volume changes and subsequent high degree of variation. This aspect is completely lost with post analysis, where the researcher is left with saved images to identify these subjective landmarks and assess spleen size. The risk may be inability to identify landmarks and hence accurately determine a valid spleen size if the image quality is poor. This specific methodology has been consistently employed in similar experiments over the past two decades across different research groups, various sonographers and across study groups, demonstrating consistent responses to a range of stressors (e.g., apnoea, exercise, normobaric hypoxia, hypobaric hypoxia) as documented previously (Elia et al., 2021, 2019; Engan et al., 2014; Holmström et al., 2019; Holmström, Mulder, et al., 2020; Lodin‐Sundström & Schagatay, 2010; Richardson et al., 2008; Schagatay et al., 2001, 2005). Although we did not actively blind the sonographer, circumstances naturally prevented him from being privy to the group membership since we did not disclose group membership as part of our procedure. Therefore, there was no apparent reason for the sonographer to be cognizant of group membership. However, we do recognize that our current method without proper blinding is a limitation in the current investigation. Second, $S_{pO_{2}}$ was measured by finger pulse oximeter, which uses a peripheral perfusion index (Lima & Bakker, 2006). One challenge with using pulse oximetry in the field, particularly at high altitude, is that peripheral vasoconstriction due to hypoxia and cold extremities can potentially influence the accuracy of the $S_{pO_{2}}$ measurements, resulting in misinterpretations and overestimations (Luks & Swenson, 2011). Therefore, all measurements were conducted indoors, maintaining peripheral skin temperatures. In addition, as all participants were measured using the same pulse oximeter, over‐ or under‐estimations of $S_{pO_{2}}$ would occur consistently across samples. Third, we did not collect blood samples during the hyperoxic test, and thus cannot explicitly link the tonic splenic contraction to changes in circulating [Hb]. However, there is abundant evidence in the literature to conclude that [Hb] changes as a direct result of the spleen volume changes in response to hyperoxia. Several laboratory studies have reliably confirmed that transient splenic contraction is closely associated with transiently elevated [Hb] in a variety of environments (Bakovic et al., 2003; Baković et al., 2005; Bouten et al., 2019; Schagatay et al., 2007, 2005). Also, in our recent study (Holmström, Bird, et al., 2020), we observed a negative association between resting spleen volume and resting [Hb] during the ascent, linking the tonic contraction to an increased O_2_ carrying capacity through the increase in circulating [Hb].

## 5 CONCLUSIONS

In summary, our study substantiated earlier observations that Sherpa populations residing at high altitude possess larger spleens compared to Nepali non‐Sherpa with lowland ancestry. Furthermore, we uncovered a novel aspect of this disparity: Sherpa spleen size remains unchanged when hypoxaemia is alleviated through hyperoxic breathing, indicating a blunted splenic response to hypoxia at rest in Sherpa. These findings suggest that the combination of larger spleens and a diminished splenic response to sustained hypoxia may represent adaptive traits in the Sherpa population, potentially enhancing the capacity for splenic contractions during periods of high metabolic demand or exercise at high altitude. However, the functional implications of these observations in terms of improving exercise capacity in hypoxic conditions require further investigation.

In stark contrast to the Sherpa population, our study revealed that spleen size transiently increases during hyperoxic breathing at high altitude in Nepali non‐Sherpa. This observation not only highlights a distinct splenic response compared to Sherpas but also supports previous evidence suggesting that the spleen undergoes tonic contraction during ascent to high altitude in lowlanders. Importantly, this tonic contraction appears to be an active process, stimulated by systemic hypoxia, rather than indirectly influenced by other factors present at high altitude. For lowlanders exposed to hypoxia, we propose that this active tonic contraction may confer functional benefits during the early stages of high altitude ascent by influencing haematological acclimatization through the elevation of [Hb], thereby aiding in the preservation of $C_{aO_{2}}$. These intriguing findings shed light on the dynamic interplay between the spleen and altitude acclimatization and warrant further investigation into their functional significance.

## AUTHOR CONTRIBUTIONS

Pontus K. Holmström, Taylor S. Harman, Abigail W. Bigham, and Tom D. Brutsaert contributed to the conception or design of the work. Pontus K. Holmström, Taylor S. Harman, Abigail W. Bigham, Anne Kalker, Kelsey C. Jorgensen, Kimberly T. Zhu, Bethany Steiner, Ella Hawkins, Trevor A. Day, Ajaya J. Kunwar, Nilam Thakur, Sunil Dhungel, Nima Sherpa, Erika K. Schagatay and Tom D. Brutsaert contributed to the acquisition, analysis or interpretation of data for the work. Pontus K. Holmström, Taylor S. Harman, Abigail W. Bigham, Anne Kalker, Kelsey C. Jorgensen, Kimberly T. Zhu, Bethany Steiner, Ella Hawkins, Trevor A. Day, Ajaya J. Kunwar, Nilam Thakur, Sunil Dhungel, Nima Sherpa, Erika K. Schagatay and Tom D. Brutsaert contributed to the drafting the work or revising it critically for important intellectual content. All authors read and approved the final version of the manuscript and agree to be accountable for all aspects of the work in ensuring that questions related to the accuracy or integrity of any part of the work are appropriately investigated and resolved. All persons designated as authors qualify for authorship, and all those who qualify for authorship are listed.

## CONFLICT OF INTEREST

No conflicts of interest, financial or otherwise, are declared by the authors.

## Data Availability

Data supporting this study are available upon request to the authors.
